# Successful pregnancy in a patient with pseudomyxoma peritonei following *in-vitro* fertilization using donor eggs

**DOI:** 10.4103/0974-1208.44119

**Published:** 2008

**Authors:** SA Narvekar, PK VijayKumar, N Shetty, MS Srinivas, KA Rao

**Affiliations:** Bangalore Assisted Conception Center, #6/7, Kumara Krupa, High Grounds, Bangalore - 560 001, India

**Keywords:** Donor egg, *in-vitro* fertilization, mucinous cystadenoma, pregnancy, pseudomyxoma peritonei

## Abstract

Pseudomyxoma peritonei is a rare, chronic relapsing disease with a guarded prognosis. Here, we describe such a case of a young patient presenting with primary infertility, who conceived following in-vitro fertilization with donor egg and had a successful pregnancy outcome. Literature regarding fertility and pregnancy outcome in this condition is reviewed.

## INTRODUCTION

Pseudomyxoma peritonei (PMP) is a rare, chronic and poorly understood disease. It is characterized by mucinous ascites and a protracted clinical course with multiple recurrences despite surgery and/or chemotherapy. There is limited data available addressing the reproductive prognosis in these patients. Also, the course of pregnancy in these patients is largely unknown. Only three pregnancies in patients with PMP have been reported to date.[1,2]

Here, we present a case of a young girl, with PMP secondary to a mucinous cystadenoma of the ovaries, who presented with primary infertility and had a successful pregnancy outcome, having conceived with *in-vitro* fertilization (IVF), using donor eggs.

## CASE REPORT

A 25-year-old patient, presented to us with primary infertility since four years. She had undergone an appendectomy at the age of 14 years and a laparotomy at the age of 20 years for acute abdomen secondary to intestinal obstruction. There were no records available of the first surgery. The intraoperative findings of the second surgery revealed jelly-like material filling the peritoneal cavity suggestive of pseudomucinous peritonei. Both ovaries were replaced by huge multiloculated cystic masses 10 × 10 cm each, densely adhered to the omentum and the sigmoid colon. There was a constriction band at the ileocaecal junction that was resected and intestinal continuity reestablished. The ovarian masses were suboptimally debulked. Both tubes were reported to be distorted and atrophic. She did not receive intraperitoneal chemotherapy as there were no facilities available. Histology revealed a benign mucinous cystadenoma of both ovaries. After the surgery, she was lost for follow-up.

At presentation to us, she had regular menstrual cycles, her FSH and LH levels being 12.1IU/L and 10.8 IU/L respectively. Other than her fertility concerns, she was asymptomatic. Transvaginal scan revealed a bulky uterus with heterogeneous multiloculated cystic masses 10 × 10 cm each, in both adnexae. Normal ovarian tissue could not be demonstrated. CA125 and CEA were normal. The patient was referred to a gynecological oncologist, who advised revision laparotomy in view of probable recurrence of the disease, but she refused. Hysterosalpingogram confirmed the bilateral tubal block. Her husband's semen parameters were normal.

Options of controlled ovarian hyperstimulation could not be considered due to the presence of persistent large cysts in both ovaries and the inability to visualize normal ovarian tissue. In such a situation, to fulfill her desire to have a child, we counseled the couple to opt for an IVF with donor oocyte. The endometrial preparation consisted of luteal downregulation with 0.5 ml of leuprolide acetate (Lupride® Sun Pharmaceutical Halol, Gujarat India Ltd), followed by sequentially increasing doses of oral estradiol valerate (Progynova®; Schering, Berlin, Germany), for a period of twelve days. Injectable progesterone 50 mg (Gestone® Ferring Pharmaceuticals Pvt Ltd, Powai, Mumbai, India) was administered daily from the day of the oocyte pickup. She had two, grade A, 8-cell stage embryos transferred. The dose of progesterone was increased to 100 mg on the day of embryo transfer and continued till 12 weeks of pregnancy. She conceived in the first cycle. The antenatal period was uneventful. The masses were persistent during the pregnancy and did not increase in size. She underwent a caesarean section for failed induction and delivered a healthy baby weighing 3 kg. Peroperatively, there were dense adhesions between the anterior abdominal wall and the uterus. The bladder was pulled up and was densely adherent to the anterior uterine wall.

There was an inadvertent injury to the bladder, which was repaired and she had an uneventful recovery. She is now 3-months postnatal. On rescan, the ovarian cysts were persistent. She has been referred to a gynecological oncologist for further management.

## DISCUSSION

The term PMP was coined by Werth in 1884[3] to describe massive intraperitoneal accumulation of gelatinous pseudomucin.[4] This condition may be associated with benign or malignant lesions of the appendix or ovary and rarely pancreas, fallopian tubes and intestines. It preferentially affects women in the age range of 29–76 years with a peak at 53 years.[2] The patients with PMP usually present with nonspecific complaints namely – nausea, fatigue, abdominal pain, distension or a mass in the abdomen – and the diagnosis is usually made accidentally, at surgery. In advanced stages, it may lead to fistula formation and adhesions, with partial or complete bowel obstruction.[1]

The treatment of PMP primarily remains aggressive surgical debulking, which may have to be done repeatedly, due to its recurrent nature. Postoperative adjuvant therapy includes the use of intraperitoneal 5-fluorouracil, mitomycin-C and cisplatin.[5] The prognosis is extremely guarded with 5 and 10-year survival rates of 50% and 20%, respectively.[2]

A pubmed search for the terms ‘infertility’ and ‘pregnancy in PMP’, revealed only four case reports,[6–9] probably because of its rarity and the poor prognosis. Hales *et al*.,[6] reported a case of secondary infertility attributed to PMP caused by ruptured mucocele of the appendix and resection of the tumor and visible mucinous ascites resulted in spontaneous conception. They hypothesized that secondary infertility was caused by significant peritoneal inflammation and inhibition of sperm–oocyte interaction from the ascites. Bocca *et al*., reported a case of primary infertility, scheduled for a laparoscopic cornual detachment of the hydrosalpinx prior to IVF. On laparoscopy, besides the hydrosalpinx, a thin layer of mucus was found covering the uterus and the tube, which on biopsy proved to be PMP, the origin of which was later found to be a low-grade mucinous appendiceal neoplasm. They concluded that malignancies, although rare, should always be part of the differential diagnosis of external causes of tubal disease.[7] Niwa *et al*., reported a case of PMP secondary to ovarian mucinous cystadenocarcinoma, who conceived naturally, twice, after surgical clearance and postoperative adjuvant chemotherapy.[8] A case of acute abdomen secondary to small bowel obstruction in a patient at 34 weeks of pregnancy with PMP secondary to mucinous cystadenoma of the appendix has also been reported.[9]

In this case report, we wish to highlight that although patients with PMP have a guarded long-term prognosis, their desire to have a child needs to be addressed. Presence of the pseudomucin in the peritoneum and the distorted tubo-ovarian relation, secondary to the dense adhesions, may impair fertility. Natural conception can occur after surgical clearance and chemotherapy. IVF may be the only option wherein corrective surgery is not possible due to dense adhesions. In our case, the presence of persistent multiloculated cysts in both ovaries and the absence of demonstratable normal ovarian tissue made controlled ovarian hyperstimulation impossible. Hence, we counseled her to opt for donor eggs.

During pregnancy, complications related to PMP, like bowel obstruction, fistula formation may occur. Dense adhesions should be anticipated at caesarean section.

To the best of our knowledge, this is the first case report of a patient with PMP, who conceived with IVF using donor eggs. The case emphasizes that pregnancies are possible in impossible situations like PMP, through ART.
